# The Impact of the Position of the Humeral Head Relative to the Glenoid on Iatrogenic Fractures During Shoulder Dislocation Reduction

**DOI:** 10.3390/medicina60111816

**Published:** 2024-11-05

**Authors:** Zeki Gunsoy, Sinan Oguzkaya, Gokhan Sayer, Fatih Golgelioglu, Mustafa Dinc, Abdulhamit Misir

**Affiliations:** 1 Department of Orthopedics and Traumatology, Bursa City Hospital, 16110 Bursa, Turkey; zekigunsoy@gmail.com (Z.G.); gkhnsyr38@gmail.com (G.S.); drindianster@gmail.com (M.D.); 2 Department of Orthopedics and Traumatology, Elazig Fethi Sekin City Hospital, 23280 Elazig, Turkey; fatihgolgelioglu@gmail.com; 3 Department of Orthopedics and Traumatology, Bahcesehir University Medical Park Goztepe Hospital, 34730 Istanbul, Turkey; misirabdulhamitmd@gmail.com

**Keywords:** complications, shoulder, fracture, humerus, glenoid

## Abstract

*Background and Objectives*: The aim of this study was to investigate the relationship between the position of the humeral head relative to the glenoid and the occurrence of iatrogenic surgical neck fractures of the humerus during anterior glenohumeral dislocation reductions. *Materials and Methods*: Patients with first-time anterior shoulder dislocations without generalized joint hyperlaxity were included. The humeral head displacement ratio was calculated as the distance between the medial border of the humeral head and the anterior glenoid rim divided by the diameter of the humeral head. Demographic data, concomitant tuberculum majus fractures, and the deltoid tuberosity index were recorded. Patients were divided into three groups: Group 1 (iatrogenic fracture development during closed reduction (CR)), Group 2 (failed CR), and Group 3 (successful reduction without iatrogenic fracture). Complicated dislocations were included in Groups 1 and 2, while uncomplicated dislocations were those in Group 3. *Results*: The study included 89 patients with a mean age of 46.44 ± 19.02 years (64 males, 25 females). Concomitant tuberculum majus fractures occurred in 37 (41.6%) cases. Iatrogenic surgical neck fractures occurred in 10 patients (Group 1), and CR was unsuccessful in 8 patients (Group 2), totaling 18 cases of complicated dislocations. Reduction without iatrogenic fracture was achieved in 71 cases (Group 3). The mean humeral head displacement ratio was higher in the complex dislocation group (92.91 ± 15.34 vs. 75.01 ± 13.80; *p* < 0.001). Complicated dislocations were more frequent in patients with tuberculum majus fractures (*p* = 0.031). Subgroup analysis showed higher humeral head displacement ratios in Groups 1 and 2 compared to Group 3 (*p* = 0.010 and *p* = 0.06, respectively). Tuberculum majus fractures were more frequent in Group 1 compared to Group 3 (*p* = 0.013), with no significant difference between Groups 2 and 3. *Conclusions*: In patients experiencing first-time traumatic anterior shoulder dislocations, a greater medial displacement of the humeral head relative to the glenoid rim significantly increases the risk of iatrogenic humeral fractures and the likelihood of unsuccessful closed reduction attempts.

## 1. Introduction

The glenohumeral joint offers the greatest range of motion in the human body but is highly susceptible to recurrent instability [1]. With an annual incidence of approximately 23.9 per 100,000 individuals, most dislocations occur anteriorly [2,3]. These dislocations are often accompanied by other injuries, such as fractures of the tuberculum majus, rotator cuff tears, or labrum tears. Despite the diverse mechanisms that can cause dislocations and associated pathologies, the primary treatment remains an urgent reduction, preferably performed under anesthesia, especially in young patients and those experiencing a first-time dislocation [4]. Although many reduction techniques have been described, there is always a risk of iatrogenic humeral fracture (IHF), regardless of the method used. IHF represents a significant complication that can arise during closed reduction attempts [5]. Initially, Gerber et al. [6] reported the challenges associated with treating this specific fracture–dislocation. Additionally, they claimed that even with careful closed reduction while under general anesthesia, perfect relaxation, and fluoroscopic control, iatrogenic fractures could still occur. Most cases of IHF necessitate internal fixation, which complicates and prolongs treatment.

In our clinical experience, we have noticed that the position of the humeral head relative to the glenoid appears to influence the risk of IHF during reduction. In particular, a more medialized humeral head seems to mechanically lock against the glenoid, making the reduction more difficult and increasing the likelihood of fractures. This observation, combined with the existing literature, has led us to hypothesize that specific anatomical factors, particularly the humeral head’s displacement, play a crucial role in the complication risk during shoulder dislocation reductions.

Previous studies have identified several risk factors for IHF, including being over the age of 40, experiencing a first-time anterior shoulder dislocation, and the presence of a greater tuberosity (GT) fracture [5]. Notably, the presence of a GT fracture and the size of the GT fragment have been shown to increase the risk of IHF during closed reduction attempts [7]. Despite this, the literature provides limited information regarding the risk of IHF during the closed reduction of glenohumeral dislocations. The identification of these risk factors will contribute to orthopedic and traumatology surgeons in the management of glenohumeral joint reductions.

This study aims to evaluate the impact of the humeral head’s position relative to the glenoid on the development of IHF during closed reduction. We hypothesize that a more medialized position of the humeral head relative to the glenoid will increase the likelihood of IHF during closed reduction procedures.

## 2. Materials and Methods

This retrospective case–control study was initiated after approval by the Bursa City Hospital institutional review board (approval number: 2023-13/6). The inclusion criteria included the following: patients admitted between January 2020 and January 2023, first-time traumatic anterior dislocation, skeletal maturity, and closed reduction attempts in the emergency department. The exclusion criteria included the following: habitual shoulder dislocation, generalized hyperlaxity or multidirectional shoulder instability dislocations other than anterior direction, and an associated glenoid fracture or initial surgical or anatomical neck of the humerus.

Fractures of the surgical or anatomical neck of the humerus were excluded due to their significant impact on humeral stability, which could interfere with the reduction process and complicate the assessment of IHF. In contrast, tuberculum majus fractures were included, as they are frequently associated with anterior shoulder dislocations and may increase the risk of IHF by causing mechanical locking of the humeral head against the glenoid. Other fractures, such as those of the tuberculum minus, clavicle, or scapular body, were not excluded, as they do not significantly affect the mechanics of reduction or the risk of IHF in the same way.

Patient records were identified using a query of the hospital’s electronic medical records system. We filtered records from patients admitted between January 2020 and January 2023 who met the following inclusion criteria: first-time traumatic anterior shoulder dislocation, skeletal maturity, and closed reduction attempts in the emergency department. The query was conducted using specific parameters to capture only those patients who fulfilled the inclusion criteria and exclusion criteria, such as habitual shoulder dislocation, generalized hyperlaxity, multidirectional shoulder instability, and glenoid fractures or preexistence fractures of the surgical/anatomical neck of the humerus. These criteria were applied during the query process to ensure accurate patient selection.

**Figure 1:**
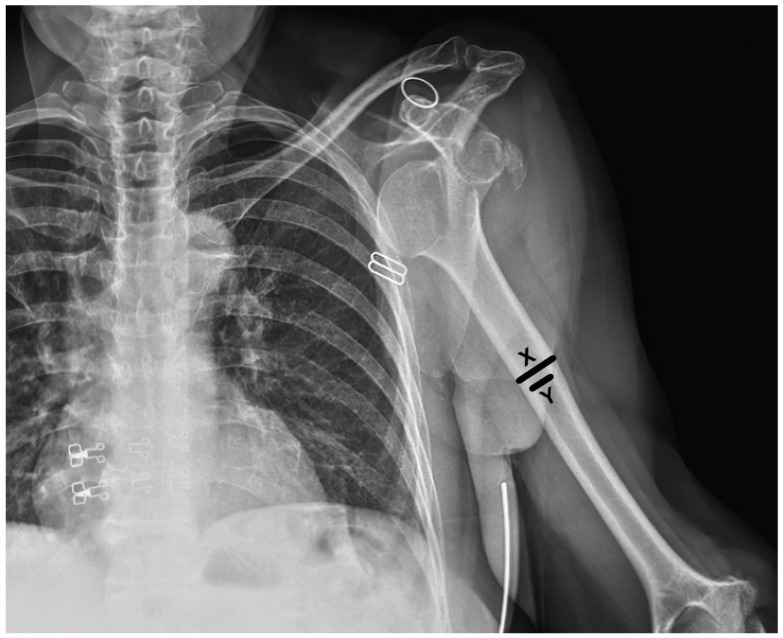
Measurement of the deltoid tuberosity index (DTI). The DTI is calculated by measuring the transverse diameter of the deltoid tuberosity at its widest point (X) and dividing by the narrowest transverse diameter (Y) of the humeral shaft at the same level.

Demographic data such as age and gender were collected from the patient records. Dislocation directions, accompanying fractures, and post-reduction IHF were recorded. The traction–counter traction method was used in all reduction attempts, and the success of the reduction and the presence of preexisting GT fractures were also documented. Also, the deltoid tuberosity index [8] was measured to assess local bone quality (Figure 1). The traction-–counter traction method was used for all reduction attempts under conscious sedation. All reduction maneuvers were performed either by an orthopedic specialist or a resident under the supervision of a specialist. The experience level of the practitioner was not specifically analyzed in this study, as all procedures were conducted under standardized conditions to minimize variability in technique. For sedation, a midazolam–fentanyl combination was administered, with midazolam dosed at 0.05–0.1 mg/kg and fentanyl at 1–2 μg/kg. In cases where this combination was contraindicated, alternative sedative agents such as propofol or ketamine were used, based on the patient’s medical history and clinical condition. Anesthesia was applied to all of the patients to ensure muscle relaxation during the reduction process. Patients were divided into three groups, as follows: Group 1 included patients who had iatrogenic fractures during CR, regardless of the success of the reduction; Group 2 included those with failed CR attempts without IHF; and Group 3 included patients with successful reduction without IHF. In cases where both iatrogenic fractures and failed reduction occurred, patients were classified under Group 1 due to the presence of a fracture, as it represents a more significant complication. Unsuccessful reduction attempts and patients with iatrogenic surgical neck fractures were classified together as complicated reductions (Groups 1 and 2). In contrast, patients who had received successful CR with or without previous tuberculum majus fractures were classified as having “uncomplicated dislocations” (i.e., Group 3).

The humeral head position relative to the glenoid in the dislocated position was evaluated using anteroposterior (AP) radiographs obtained in the emergency department before reduction. To minimize variability in the measurements, all X-rays were taken under standardized conditions with the patient in a supine position and their arm in a neutral position.

**Figure 2:**
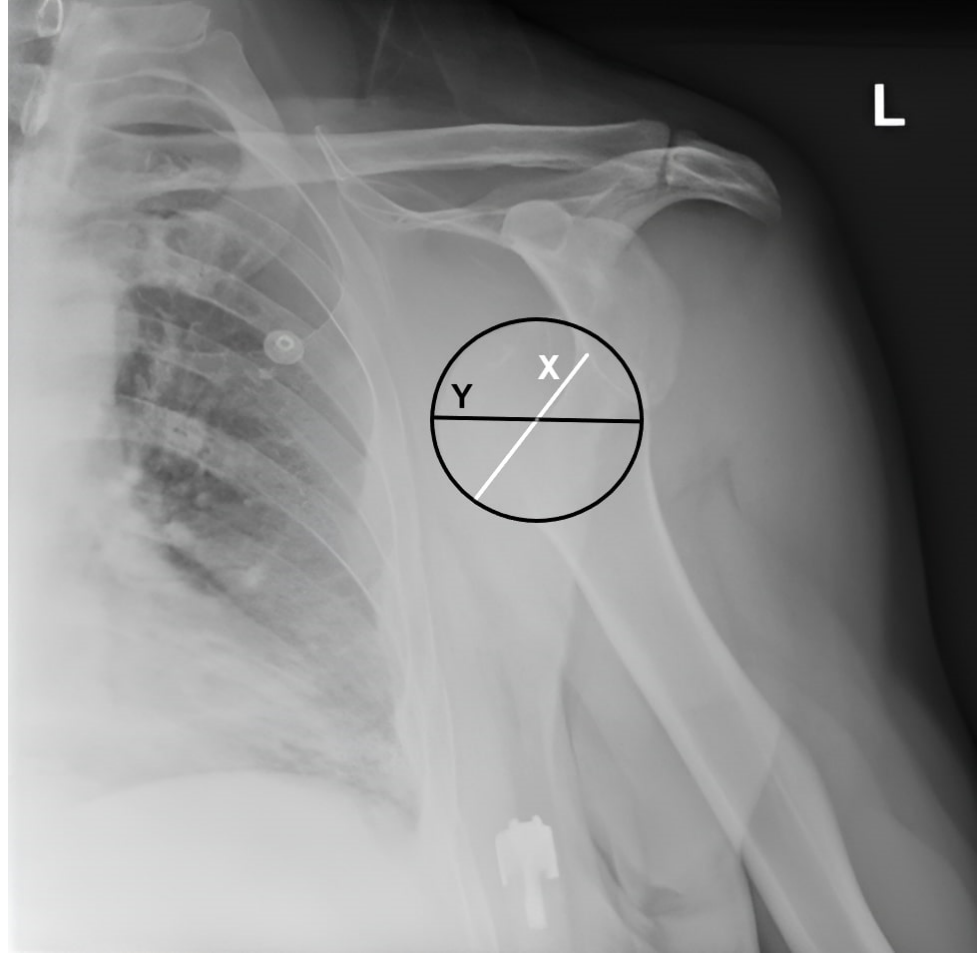
Measurement of humeral head displacement ratio. The humeral head displacement ratio is calculated as the distance between the medial border of the humeral head and the anterior glenoid (X) border divided by the humeral head diameter (Y) and multiplied by 100, L: Left.

The humeral head position relative to the glenoid in the dislocated position was evaluated, and the ‘humeral head displacement ratio’ was calculated as the distance between the medial border of the humeral head and the anterior glenoid rim divided by the diameter of the humeral head on AP radiographs before reduction (Figure 2).

### Statistical Analysis

The mean, standard deviation, median, lowest and highest value, frequency, and ratio were used to present descriptive statistics. The Shapiro–Wilk test was performed to evaluate the variable distribution. The chi-square and Fischer exact tests were performed to compare independent qualitative data. The Mann–Whitney U and Kruskal–Wallis tests were performed to compare independent quantitative data. Given the presence of multiple comparisons among the three groups, post-hoc analyses were performed using pairwise comparisons. To control for the potential increase in Type I error due to multiple comparisons, we applied the Bonferroni correction to adjust the alpha level. The corrected alpha level was used to determine statistical significance in all post-hoc analyses. A *p*-value of less than 0.05 was considered statistically significant unless adjusted by the Bonferroni correction. All statistical analyses were performed using IBM SPSS version 22 for Windows (IBM Corp., Armonk, NY, USA).

## 3. Results

Initially, a total of 110 patients met the inclusion criteria based on the electronic medical record query. However, 21 patients were excluded for the following reasons: 8 patients had recurrent shoulder dislocations, 5 had associated glenoid fractures, 4 had surgical or anatomical humeral neck fractures, 3 had multidirectional shoulder instability, and 1 patient had generalized hyperlaxity. After applying these exclusion criteria, a total of 89 patients were included in the final analysis. The mean age of the patients was 46.44 ± 19.02. There were 64 male (71.9%) and 25 female (28.1%) patients. A concomitant tuberculum majus fracture was observed in 37 (41.6%) cases. Iatrogenic surgical neck fracture occurred in 10 (Group 1) patients, and CR was unsuccessful in 8 patients (Group 2). Altogether, 18 patients were considered to have complicated dislocations (Groups 1 and 2). Reduction was achieved without iatrogenic fracture in 71 cases (Group 3). There was no significant difference in age, gender, deltoid tuberosity index (DTI), or frequency of tuberculum majus fracture before reduction (Table 1).

The mean humeral head displacement ratio was significantly higher in the complicated dislocation group (92.91 ± 15.34 vs. 75.01 ± 13.80; *p* < 0.001). The frequency of complicated dislocations was significantly higher in patients with tuberculum majus fracture–dislocations (*p* = 0.031). The humeral head displacement ratio was higher in Group 1 and Group 2 compared to Group 3 (*p* = 0.010 and *p* = 0.060, respectively). Tuberculum majus fracture frequency was higher in Group 1 compared to Group 3 (*p* = 0.013), while no difference was observed between Groups 2 and 3 (*p* > 0.05).

## 4. Discussion

The most important finding of this study was that a greater medial displacement of the humeral head relative to the glenoid rim significantly increases the risk of iatrogenic humeral fractures and the likelihood of unsuccessful closed reduction attempts in patients experiencing first-time traumatic anterior shoulder dislocations. This underscores the critical role of the humeral head’s position in predicting complications during the closed reduction of anterior shoulder dislocations. Our results indicate that when the humeral head is more medially displaced, it may become mechanically locked against the glenoid, increasing the difficulty of achieving a successful reduction without causing additional injury. This locking mechanism is particularly pronounced when a concomitant tuberculum majus fracture is present, further complicating the reduction process.

The absence of anterior rim glenoid fractures in cases where the humeral head was mechanically locked against the glenoid rim may be attributed to the controlled reduction methods used in our study. Conscious sedation ensured optimal muscle relaxation during reduction, reducing the likelihood of excessive force being applied. Moreover, the traction–counter traction method helped to distribute forces more evenly during reduction, potentially preventing glenoid fractures.

The presence of GT fractures was found to be a significant factor in complicated dislocations, consistent with prior studies identifying them as a risk factor for iatrogenic fractures during closed reduction [8]. In the current study, iatrogenic fractures were more frequent when GT fractures were present. However, cases of further displacement or fragmentation of GT fractures after reduction were treated as separate entities and were not classified as IHF, as we consider them to be different from surgical neck fractures. Atoun et al. [5] reported a 5.4% incidence of post-reduction humeral fractures in a retrospective analysis of 92 first-time shoulder dislocations in patients over 40, with the rate increasing to 26% in those with concomitant large tuberosity fractures. Similarly, Guo et al. [7] found that larger GT fragments were associated with a higher risk of IHF. Our findings align with these studies [7,9], as we observed a higher frequency of IHF in the presence of initial GT fractures. The frequency of IHF in our study exceeded that reported in the previous literature (11.2%), which may be attributed to the fact that some patients had unsuccessful reduction attempts at other hospitals before being referred to our tertiary centre. In contrast to common expectations, we did not observe a relationship between IHF and patient age or bone quality.

We acknowledge, however, that our study cannot definitively distinguish between occult fractures of the surgical or anatomical neck and complete fractures in the context of anterior shoulder dislocations. Occult fractures may occur at the time of dislocation and remain undetected without advanced imaging techniques such as a CT scan. While all of the patients underwent X-ray evaluation, a CT scan could have helped differentiate between fractures that occurred during the dislocation event and those that may have occurred during the reduction process. Therefore, this study can only report the visible incidence of surgical neck fractures, and future research should include CT imaging to improve the accuracy of fracture detection in similar cases.

This study found that the high rate of tuberculum majus fractures in Group 1 is not solely due to it being an independent parameter but also because it acts as a mechanical factor that complicates the reduction process. These fractures cause the humeral head to mechanically lock against the glenoid, increasing the difficulty of reduction and the risk of iatrogenic humeral fractures. Therefore, the presence of tuberculum majus fractures contributed to the higher incidence of complicated reductions in our series.

In their study, Wronka et al. [10] reported a 94% success rate in the closed reduction of anterior dislocation with GT fracture. Yuan et al. [11] retrospectively analyzed 29 patients with 30 anterior fracture–dislocations, and they proposed a treatment algorithm and concluded that all fracture–dislocations could be reduced in the ER unless the humeral head is locked under the glenoid. When the humeral head is locked under the glenoid, the reduction maneuver may cause a surgical neck fracture. We tried to formalize the position of the humeral head relative to the glenoid. We believe that the higher the humeral head displacement rate, the higher the risk of developing IHF due to the “locking” mechanism of the humeral head, with or without a GT fracture. There is no consensus about the reduction technique for anterior shoulder dislocation. A recent systematic review and meta-analysis showed that traction–counter traction methods were less painful, with similar complication rates and reduction success compared to the other methods [12]. We know that reduction maneuvers may affect the success of reduction and IHF. In our clinic, all patients received the same reduction maneuver; therefore, the effect of the reduction maneuver was not analyzed as a potential risk factor for IHF.

The decision to perform shoulder dislocation reductions under anesthesia is increasingly supported, especially in young patients and first-time dislocations [13]. Our findings suggest that patients with a higher humeral head displacement ratio, particularly those with concomitant tuberculum majus fractures, are at increased risk for iatrogenic humeral fractures during closed reduction. In such cases, we recommend that closed reduction be performed in the operating theatre under general anesthesia, as this ensures optimal muscle relaxation and reduces the risk of applying excessive force. Additionally, in cases where the reduction appears mechanically challenging, early consideration for open reduction may be warranted to avoid further complications. General anesthesia not only facilitates smoother and less painful procedures but also improves the success rate and minimizes complications. In younger patients, muscle spasm and tissue resistance can further complicate reductions, making anesthesia even more crucial [14,15]. In this study, all reductions were performed under conscious sedation, which may have influenced the incidence of iatrogenic fractures.

Reduction failure is a rare condition in glenohumeral dislocations. It is usually associated with mechanical block due to the interposition of soft tissues or fracture fragments [16]. In the literature, most of the reports related to irreducibility were case reports. In this study, we evaluated a relatively high number of patients. In addition, we also showed that the increase in the medialization of the humeral head relative to the glenoid with the humeral head displacement ratio we defined was associated with irreducibility and humeral neck fracture. These findings support the interposition effect in the literature but also indicate that the possibility of complications may increase, possibly due to the high energy of the dislocation formation and the high energy required for reduction.

This is the first study to evaluate the role of dislocated humeral head position in the risk of IHF. In this respect, we believe that it will fill the gap in the literature. These findings have important clinical implications. Orthopedic and traumatology surgeons should carefully evaluate the humeral head displacement ratio and the presence of tuberculum majus fractures before performing closed reduction. This can help identify patients at higher risk for complications and guide the decision-making process regarding the need for surgical intervention. Future researchers should consider exploring the potential benefits of alternative reduction techniques, such as Kocher’s maneuver, in patients with high humeral head displacement ratios to determine if different methods can reduce the risk of iatrogenic fractures. Additionally, prospective studies involving larger cohorts could provide more robust data to validate the findings of this study. Finally, developing a standardized protocol for the assessment and management of anterior shoulder dislocations with a focus on minimizing complications could significantly enhance patient outcomes.

This study has several limitations that should be noted. First, the radiologic evaluation was based only on AP X-rays obtained in the emergency department. The patient’s position, such as slight oblique or lateral tilting, arm placement, and body habitus (e.g., weight or clothing), may have introduced variability in our measurements. Despite efforts to standardize conditions, these factors could still have influenced the accuracy of the measurements. Furthermore, we acknowledge the lack of CT imaging, which could have been useful in detecting occult fractures of the surgical or anatomical neck. The bone quality of patients may also influence the fracture risk. We tried to evaluate the bone quality with the deltoid tuberosity index. Since all patients received the traction-counter–traction method, we were unable to compare the impact of different reduction maneuvers. This limited our ability to generalize our findings to other reduction techniques. Another limitation of this study is that the experience level of the practitioners performing the reduction was not taken into account. Lastly, the patient population was relatively low, so reliable analyses are needed to determine independent risk factors for IHF.

## 5. Conclusions

This study emphasizes the need for a thorough pre-reduction assessment of the humeral head position relative to the glenoid rim. Recognizing the increased risk associated with greater medial displacement, particularly in patients with concomitant tuberculum majus fractures, can improve patient outcomes by minimizing the incidence of iatrogenic fractures and unsuccessful reduction attempts. We recommend that in cases with a high humeral head displacement ratio, closed reduction should be performed in the operating theatre under general anesthesia to reduce the risk of complications. Early consideration for open reduction may also be necessary in cases where the reduction appears mechanically challenging. Future research should focus on developing standardized protocols for radiographic evaluation and management strategies in these high-risk cases.

## Figures and Tables

**Table 1:** Demographic data of all groups.

	Group 1 *n* = 10	Group 2 *n* = 8	Group 3 *n* = 71	*p*
Age (Years ± SD)	54.8 ± 16.1	50.5 ± 12.2	44.81 ± 19.8	0.149
Female/Male (%)	5 (50%)/5 (50%)	2 (25%)/6 (75%)	18 (25.4%)/53 (74.6%)	0.262
DTI (mean ± SD)	1.27 ± 0.45	1.48 ± 0.13	1.43 ± 0.14	0.474
Tuberculum majus fracture (n-%)	8 (80%)	4 (50%)	25 (35.2%)	0.024

DTI: deltoid tuberosity index.

## Data Availability

The data can be obtained from the corresponding author upon request.
